# Genomic regions associated with flag leaf and panicle architecture in rice (*Oryza sativa* L.)

**DOI:** 10.1186/s12864-024-11037-z

**Published:** 2024-12-18

**Authors:** Gurjeet Singh, Subroto Das Jyoti, Priyanka Uppalanchi, Rushika Chepuri, Sejuti Mondal, Chersty L. Harper, Punniakotti Elumalai, Ken Mix, Nicole Wagner, Darlene L. Sanchez, Stanley Omar PB. Samonte, Shyamal K. Talukder

**Affiliations:** 1 Texas A&M AgriLife Research Center, Beaumont, TX 77713 USA; 2 Texas State University, San Marcos, TX 78666 USA

**Keywords:** Rice, Flag leaf, Panicle architecture, GWAS, Candidate gene

## Abstract

**Background:**

Flag leaf (FL) and panicle architecture (PA) are critical for increasing rice grain yield as well as production. A genome-wide association study (GWAS) can better understand the genetic pathways behind complex traits like FL and PA.

**Results:**

In this study, 208 diverse rice germplasms were grown in the field at the Texas A&M AgriLife Research Center at Beaumont, TX, during 2022 and 2023 following Augmented Randomized Complete Block Design. After heading, eight different flag leaf and panicle architecture (FLPA) related traits were measured. GWAS analyses were performed to identify potential genomic regions associated with FLPA traits. A total of 97 quantitative trait loci (QTLs) (48 in 2022 and 49 in 2023) were distributed across all 12 chromosomes. GWAS revealed four QTLs (*qSBPP4-2*, *qFLW6-2*, *qGNPP9*, and *qGWPP2-3*) with phenotypic variation ranging from 11.7 to 22.3%. Two genetic loci were identified as multi-trait QTLs, i.e., S04_32100268 (*qFLL4-1* and *qFLA4-1*) and S04_11552936 (*qFLW4* and *qFLA4-2*) during 2022 and 2023, respectively. Additionally, these loci were further utilized to analyze candidate genes, and 65 genes were predicted in the 100-kb genomic region upstream and downstream. In silico expression analysis revealed 15 genes were expressed during the reproductive stage. These genes were associated with protein kinase, heat shock transcription factor family, sugar transporter conserved site and transcription factor bHLH95- like basic helix-loop-helix domain protein, as well as those that regulate the FLPA-related traits. *Os04g0631100* was identified as a potential candidate gene that is highly expressed during the endosperm development stage, and it is associated with an important sugar transporter protein that will be helpful in grain improvement.

**Conclusion:**

GWAS results revealed four major and two multi-trait QTLs. Expanding their candidate genes, and expression analysis provide the genetic information for molecular improvement of the FLPA-related trait in rice breeding programs.

**Supplementary Information:**

The online version contains supplementary material available at 10.1186/s12864-024-11037-z.

## Background

Over half of the world’s population relies on rice as their staple food [1], and it is a key source of calories and nutritional quality in the majority of nations with limited resources [2, 3]. Since the global population is increasing, rice production needs to be expanded to ensure food security [4] under low-input agriculture [5]. While rice is cultivated in many regions worldwide, climate change makes sustainable intensification of rice yield difficult [6]. In this context, developing strategies to improve yield contributing traits by dissecting complex traits through genome-wide association study (GWAS) is pivotal for enhancing genetic gain in rice [7]. Rice grain yield is determined from the number of panicles, grain number per panicle (GNPP), and grain weight per panicle (GWPP) [8]. These three major components are also affected by panicle length (PL), number of primary branches per panicle (PBPP), and number of secondary branches per panicle (SBPP). Leaf shape influences yield potential by altering light interception and photosynthetic rates [9]. The flag leaf (FL) is the source of more than half of the carbohydrates accumulated during rice grain formation [10]. Flag leaf and panicle architecture (FLPA) related traits have major implications for increasing rice grain yield and production [11–14]. Hence, pre-breeding for FL and PA is important for understanding the genetic and molecular basis of rice yield. Improving flag leaf length (FLL), flag leaf width (FLW), flag leaf area (FLA), and PA will be crucial in increasing the yield potential of rice cultivars to meet the increasing demand [15].

Several quantitative trait loci (QTLs)/genes regulate the FLPA-related traits in rice. Over the years, researchers have identified multiple genes controlling panicle-related traits in rice. For example, *DST* (*DROUGHT AND SALT TOLERANCE*) regulates PL, PBPP, and SBPP [16, 17], *OsGRF4* positively regulates grain shape and PL [18], *OsLG1* controls PA [19], *lt1* (*low tiller 1*) affects panicle branching [20], *OsSPL14* increases grain productivity [21], and *IPA1* (*Ideal Plant Architecture 1*) modulates GNPP and tiller number [22]. In addition, these genes have been linked to several pathways, such as the ubiquitin-proteasome pathway, G-protein signaling pathway, MAPK signaling pathway, and the cytokinin signaling pathway [23]. Protein kinase and the protein heat shock transcription factor family play an important role during the panicle development stage [24, 25]. The sugar transport protein family and bHLH (basic helix-loop-helix) protein family regulate the FL traits and help in different plant development stages [26–29]. Protein kinase and sugar transport proteins play significant role in the development of FLPA related traits, which ultimately helps to improve the rice yield. Although significant progress has been achieved in identifying regulatory genes, identifying QTLs followed by validating and developing molecular markers are more feasible for crop improvement programs due to the quantitative nature of these traits. Interestingly, numerous QTLs that control PA in rice have been identified. For example, *qPN1*controls multiple panicle traits including PL, GNPP [30], *qPL6* on chromosome 6 covering 25 kb region controls PL [31], *qOPW11*controls panicle weight [32], *qTSN12.1* and *qTSN12.2* increase total number of spikelet by increasing the branching in a panicle [33], *qSBN7* controls SBPP [34], and *qSPP2.2* regulates spikelet per panicle [35]. Likewise, several studies identified QTLs controlling FL morphology in rice. Some examples of such QTLs include *qFLW7.2* for FLW [31], *qFLL9* for FLL [36], *qFL1* for overall flag leaf size [11], *qFLL6.2* for FLL [37], *qFLW4* for FLW and *qFLAG5* for flag leaf angle [38]. Recently, GWAS has been more convenient for studying FLPA-related traits from diverse germplasm accession than traditional bi-parental mapping. GWAS offers a more convenient and potentially more powerful approach to studying genetic traits by utilizing existing naturally occurring genetic variation within a diverse set of accessions, rather than requiring the extensive time to development of mapping populations.

Furthermore, sequencing technology is improving while its cost is decreasing substantially. Consequently, GWAS has been used frequently in candidate gene mining and marker identification for complex traits [39]. Fortunately, many studies have conducted GWAS on FLPA-related traits. For instance, GWAS conducted on diverse rice accessions identified 153 QTLs governing PA-related traits [14], 29 QTLs controlling panicle morphological traits [40], multiple marker-trait associations for panicle morphology and grain structures [41], four QTLs controlling PL [42], multiple QTLs controlling FL size through SNP and haplotype-based GWAS [43], and 64 QTLs identified for FL-related traits [12]. The results of these studies help to develop molecular markers for marker-assisted selection, which will speed up the selection process. For example, the findings of a GWAS was used to develop markers for PA-related traits [44]. Identified potential candidate genes were used for expression analysis by RiceXPro to better understand the gene function and see the expression level under specific tissues of plants [45]. Multiple results have been reported using RiceXPro to identify the potential candidate genes at various growth stages for impotent traits, such as agronomic, root-related, and reproductive [46–48]. Besides, genomic variants identified from GWAS results can be used as a starting point in genome editing programs to develop desirable changes in the rice genome.

Due to the complex inheritance nature of these FLPA-related traits, the environment plays a significant role in the phenotypic variation of these traits. Therefore, it is crucial to study these important traits in the target environment to understand the genotype-environment interaction better. To date, all the GWAS studies that were conducted had focused either on FL or PA related traits. In this context, the current study was done to perform a combined GWAS on FLPA traits to identify suitable markers associated with these important traits for rice improvement. In this present research, we studied eight FLPA-related traits, namely FLL, FLW, FLA, PL, PBPP, SBPP, GWPP, and GNPP, in 208 diverse genotypes over two years in the field to perform GWAS. We aimed to identify marker-trait associations and candidate genes that govern FLPA-related traits in rice. The results of these investigations will help design markers for rice molecular breeding programs. Additionally, the research will shed light on the possible genomic regions that can be modified through genome engineering techniques for desirable changes in the future. Similarly, potential germplasm will be identified and integrated into the breeding program for target trait improvement.

## Materials and methods

### Plant materials and experiment design

A total of 208 (including four check varieties) diverse rice accessions, including *indica* and *japonica* cultivars, landraces, and inbred lines [49], were phenotyped at the Texas A&M AgriLife Research Center at Beaumont, TX, USA. The germplasms were grown following Augumented Randomized Complete Block Design in six blocks, with four checks (C1 = Antonio, C2 = Cheniere, C3 = Cocodrie, and C4 = Presidio) as replication in each block, in 2022 and 2023. During both years, each germplasm line was direct-seeded into three rows of 2.43 m each, using a six-row planter. The row-to-row distance of the planting was 0.28 m. A maintained and recommended package of cultural practices was followed to raise a healthy crop.

### Phenotypic data collection and analysis

The data were recorded for flag leaf and panicle architecture (FLPA)-related traits using five randomly selected plants from the three rows of each germplasm line. The FLPA traits included flag leaf length in cm (FLL), flag leaf width in cm (FLW), flag leaf area in cm^2^ (FLA), panicle length in cm (PL), primary branches per panicle (PBPP), secondary branches per panicle (SBPP), grain weight per panicle in gram (GWPP), and grain number per panicle (GNPP). All phenotypic data were analyzed in RStudio using the “Agricolae” (Team 2013) and “augmented RCBD” packages [50]. A statistical normality test was performed using the Shapiro-Wilk test in RStudio with the ‘dplyr’ package. Pearson’s correlation coefficient analysis was carried out using the “Corrplot” package built in RStudio [51]. Using the replicated data of four checks, which corresponded to all the measured traits, adjusted means were calculated by “augmented RCBD” [50], which were then used for GWAS analysis.

### Molecular marker data and population structure

The marker information of the population was earlier reported [49, 52]. In brief, a total of 1,075,302 sequence-based SNP markers were used at the start of this study. The raw marker data were filtered to eliminate SNPs with > 50% missing data and minimum allele frequency (MAF) < 5%, reducing the raw marker data to 1,075,302 SNPs, and imputation was carried out using BEAGLE V4.0 [53]. The selected markers were imputed and filtered using TASSEL 5.2.61 [54], where SNPs having low-quality clustering and MAF < 5% were removed, and 854,832 SNPs were finally selected for further association analysis. All marker data are available on Dryad (10.5061/dryad.4qrfj6qbs). STRUCTURE v2.3.4 software was used to categorize sub-populations to observe the population structure in the rice panel used in this study based on the admixture model with correlated allele frequency [55]. The factors that may cause the false trait-SNP associations, i.e., population structure (Q) and genetic relatedness (K), were controlled using principal component analysis (PCA) and kinship matrix, respectively. Previous research using the same population as this experiment has calculated the PCA kinship matrix [56] and linkage disequilibrium (LD) decay [49, 52, 57]. Of the entire genetic variation, 56.7% has been described by the first four main principal components (PC). The first PC separated the *indica* from the *japonica* rice, explaining 38.9% of the variation. The second PC, contributing to 12.0% of the variation, further classified the *japonicas* into temperate and tropical subpopulations. There were admixed rice accessions between these three main subgroups [49, 52, 57]. To assess the required resolution for association mapping, LD decay, defined as the distance at which the mean r^2^ decreased to half its highest value, was estimated using TASSEL 5.2.61 [54]. The average genome-wide LD decay of the population has been calculated to be approximately 150,000 bp [49, 52, 57].

### Genome-wide association analyses, candidate gene identification, and expression analysis

The adjusted mean of both years was used to perform GWAS analysis. The small impact of genetic loci in complex polygenic attributes can be captured using multi-locus approaches, which have recently gained popularity and feasibility. So, we used these multiple methods, taking advantage of the algorithmic relevance of distinct models and supporting their findings one after the other. The R package mrMLM v5.0 (https://cran.r-project.org/package=mrMLM) was used with default parameters to perform GWAS analysis using the multi-locus approach [58]. There are six multi-locus models in the package, including mrMLM [59, 60], FASTmrMLM [61], FASTmrEMMA [62], pKWmEB [63], pLARmEB [64] and ISIS EMBLASSO [65]. When estimating QTLs, the FASTmrEMMA model outperforms mrMLM in speed, statistical power, and accuracy [61]. In comparison to mrMLM and FASTmrEMMA, ISIS EM-BLASSO has the best robustness and accuracy for detecting significant associations [65]. The pLARmEB model integrates least angle regression with empirical Bayes, allowing for a more accurate estimate of significantly linked QTLs in polygenic backgrounds [64]. QTLs with LOD scores greater than 3.00 were considered important (highlighted in pink on the Manhattan plot). Furthermore, QTLs found in at least two models were considered to be reliable.

QTLs (marker sequences) strongly linked with FLPA (flag leaf and panicle architecture) traits were searched against the *O. sativa* japonica genome assembly IRGSP-1.0 Online Web Resource Ensembl Plants (https://plants.ensembl.org/Oryza_sativa/Info/Index/Tools/Blast) using BLASTn with standard parameters to locate potential candidate genes. Furthermore, we investigated the physical positions of the SNP marker’s upstream and downstream 100 kb regions and identified genes inside each locus. Further identified candidate genes were used for in silico expression analysis by RiceXPro (Rice Expression Profile Database https://ricexpro.dna.affrc.go.jp/) during the entire growth period and reproductive stage of the crop [45]. We used different crop stages for expression analysis, including spatio-temporal gene expression of various tissues/organs throughout the entire growth in the field, gene expression profile during reproductive organ development, grain gene expression profile at the early developmental stage, and embryo and endosperm gene expression profile at ripening stage [66, 67].

## Results

### Phenotypic variation for FLPA

**Fig. 1:**
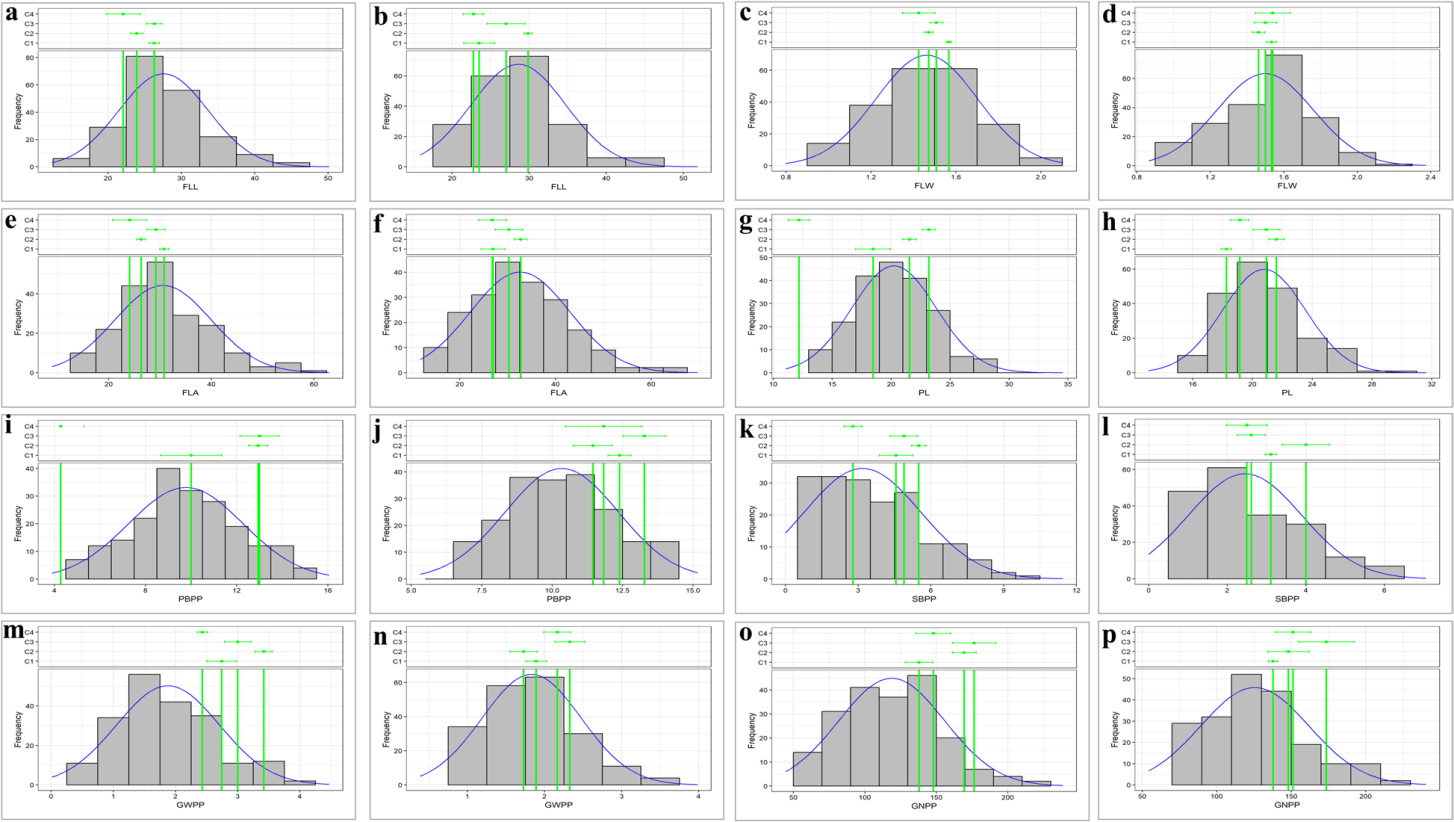
Phenotypic data distribution of diverse rice accessions: flag leaf length in 2022 (**a**), and during 2023 (**b**); flag leaf width during 2022 (**c**), and during 2023 (**d**); flag leaf area during 2022 (**e**), and during 2023 (**f**); panicle length during 2022 (**g**), and during 2023 (**h**); primary branch per panicle during 2022 (**i**), and during 2023 (**j**); secondary branch per panicle during 2022 (**k**), and during 2023 (**l**); grain weight per panicle during 2022 (**m**), and during 2023 (**n**); grain number per panicle during 2022 (**o**), and during 2023 (**p**)

**Fig. 2:**
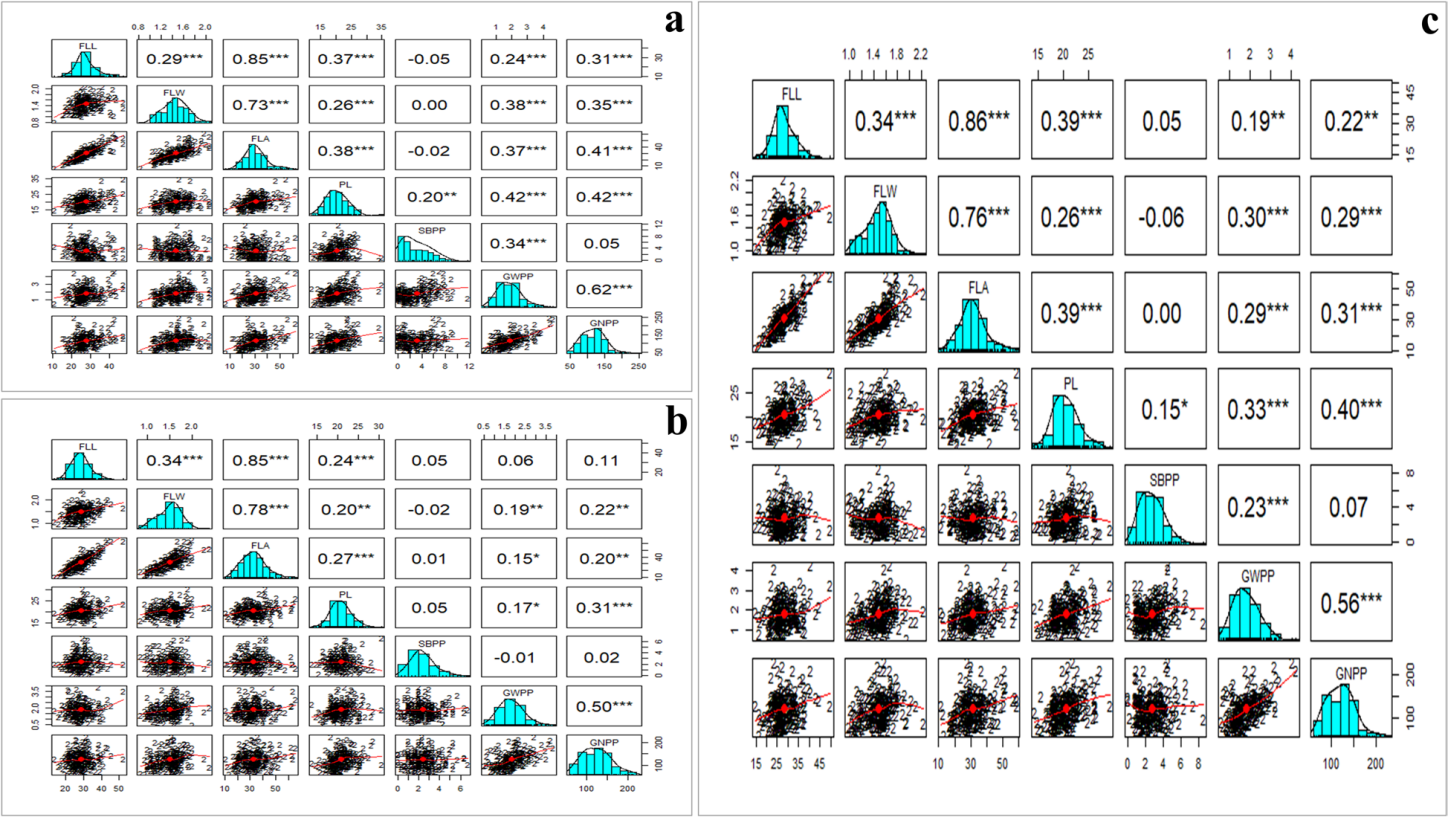
Correlation plot of diverse rice germplasm accessions for flag leaf and panicle architecture related traits: year 2022 (**a**), year 2023 (**b**), and pooled data of both years (**c**)

All traits showed normal phenotypic distribution in 2022 and 2023 (Fig. 1). The Shapiro-Wilk test was used to assess statistical normality, revealing that the *p*-values for FLW and PBPP were greater than 0.05, while the p-values for other traits (FLL, FLA, PL, SBPP, GWPP, and GNPP) were almost equal to 0.05. Year-wise analysis of variance (ANOVA) showed significant variation in all the studied traits except PBPP and GWPP in 2023 (Table 1). Considering year as environment and as a factor, genotypes were significantly different for all traits except PBPP, and environmental influence was significant for all traits (Table 1). All traits exhibited non-significant differences in the pooled ANOVA at the block level; likewise, the genotype x environment interaction indicated non-significant differences except for PL and SBPP. Descriptive statistical analysis was performed for FLPA-related traits, and a wide range of variability was observed during 2022 and 2023 (Table 2). The average of FLL was 27.56 and 28.58 cm, FLW was 1.46 and 1.50 cm, and FLA was 30.57 and 32.62 cm^2^ in 2022 and 2023, respectively. For panicle-architectural traits, the mean PL was 20.24 and 20.78 cm, PBPP was 9.77 and 10.34, and SBPP was 3.18 and 2.45. GWPP was 1.88 and 1.83 g, and GNPP was 118.71 and 125.51 in 2022 and 2023, respectively. Among all the studied traits, the flag leaf traits exhibited lower coefficients of variation (CV) than the panicle traits. The lowest was observed for FLW (6.13 cm in 2022 and 10.57 cm in 2023), while the highest CV was observed for SPBB (36.74 in 2022 and 31.31 in 2023). Skewness showed significant differences for all the traits except FLW and PBPP, whereas kurtosis also showed significant differences for FLL, FLA, and PL during both years (Table 2). Pearson’s correlation coefficients were carried out for independent years and the mean data of both years for all traits (Fig. 2). FLA had a positive and significant correlation with FLL and FLW in 2022 (*r* = 0.85 and *r* = 0.73), 2023 (*r* = 0.85 and *r* = 0.78), and the average of both years are *r* = 0.86 and *r* = 0.76, respectively. GNPP showed significant correlations with GWPP of *r* = 0.62, *r* = 0.50, and *r* = 0.56 in 2022, 2023, and with the average data of both years, respectively.

**Table 1:** Analyses of variance for flag leaf and panicle architecture-related traits of diverse rice germplasm

**Source of variation**	**df**	**Year**	**Mean Squares**							
			**FLL**	**FLW**	**FLA**	**PL**	**PBPP**	**SBPP**	**GWPP**	**GNPP**
**ANOVA based on single year **										
Block (ignoring Genotypes)	5	2022	23.91	0.02*	35.41	27.29**	4.43	8.80**	0.58*	180.57
		2023	13.48	0.03	34.72	7.56*	1.78	1.47	0.27	473.08
Treatment (eliminating Blocks)	207	2022	34.24**	0.05**	79.56**	14.35*	6.05	5.78**	0.75**	1433.54
		2023	33.13*	0.07*	98.27**	6.87**	3.48	1.68*	0.40	1264.02
Treatment: Checks	3	2022	25.21	0.02	53.94	141.68**	100.83**	8.15**	1.05**	1949.72
		2023	64.86*	0.01	48.29	14.41**	3.80	2.81*	0.43^ns^	1363.11
Treatment: Genotype and Genotype vs. Check	204	2022	34.37**	0.05**	79.93**	12.48	4.66	5.74**	0.75**	1425.95
		2023	32.66*	0.07*	99.00**	6.76 **	3.47	1.67*	0.40	1262.56
Residuals	15	2022	9.29	0.01	19.94	5.96	4.53	1.44	0.19	970.42
		2023	15.25	0.03	29.93	1.94	4.56	0.61	0.20	1177.33
**Pooled ANOVA based on both years **										
Genotype	211	-	55.74***	0.09684***	149.1***	14.18***	5.9	4.2***	0.8751***	2251***
Block	4	-	12.5	0.02033	40.5	4.81	1.71	2.13	0.1721	288
Environment (two years)	1	-	121.97**	0.13411*	457.5***	40.19**	58.32**	66.33***	1.607**	3756*
Genotype x Environment	211	-	10.66	0.01904	25.4	7.4*	3.52	3.32***	0.2793	465
Residuals	28	-	15.7	0.01803	33	4.07	5.24	1.11	0.1737	705

*df *Degree of freedom, *FLL* Flag leaf length (cm), *FLW* Flag leaf width (cm), *FLA* Flag leaf area (cm^2^), *PL *Panicle length (cm), *PBBP* Primary branch per panicle (number), *SBPP* Secondary branch per panicle (number), *GWPP* Grain weight per panicle (g), and *GNPP* Grain number per panicle (number)

Level of significant: *P <= 0.05; **P <= 0.01, ***P <= 0.001

**Table 2:** Descriptive statistics for flag leaf and panicle architecture in diverse rice germplasm

Traits	Year	Mean	Min	Max	SE	SD	Skewness	Kurtosis	CV
FLL	2022	27.56	12.34	50.15	0.42	6.08	0.76**	4.21**	11.16
	2023	28.58	15.85	51.82	0.42	6.13	0.76**	4.42**	13.78
FLW	2022	1.46	0.80	2.10	0.02	0.24	0.08	2.93	6.13
	2023	1.50	0.86	2.37	0.02	0.26	−0.06	3.03	10.57
FLA	2022	30.57	8.89	62.97	0.65	9.38	0.58**	3.81*	14.73
	2023	32.62	11.73	69.63	0.72	10.35	0.65**	3.82*	16.93
PL	2022	20.24	11.03	34.56	0.25	3.58	0.50**	4.28**	12.14
	2023	20.78	13.06	31.61	0.19	2.77	0.54**	3.98*	6.72
PBPP	2022	9.77	3.89	16.06	0.17	2.51	0.10	2.68	21.72
	2023	10.34	5.32	15.16	0.14	2.01	0.15	2.51	20.32
SBPP	2022	3.18	0.00	11.44	0.17	2.40	0.66**	2.93	36.74
	2023	2.45	0.00	7.05	0.10	1.44	0.64**	3.01	31.31
GWPP	2022	1.88	0.01	4.47	0.06	0.83	0.60**	3.36	22.31
	2023	1.83	0.39	3.98	0.04	0.64	0.50**	3.51	24.37
GNPP	2022	118.71	44.43	238.43	2.57	37.01	0.50**	3.38	25.5
	2023	125.51	54.31	240.39	2.51	36.20	0.46**	2.94	26.83

*FLL*  Flag leaf length (cm), *FLW* Flag leaf width (cm), *FLA*  Flag leaf area (cm^2^), *PL*  Panicle length (cm), *PBBP*  Primary branch per panicle (number), *SBPP*  Secondary branch per panicle (number), *GWPP *Grain weight per panicle (g), and *GNPP*  Grain number per panicle (number). *SE *Standard error, *SD* Standard deviation, and *CV*  Coefficient of variation.

Level of significant: *P < = 0.05; **P < = 0.01

## GWAS analysis for FLPA

**Fig. 3:**
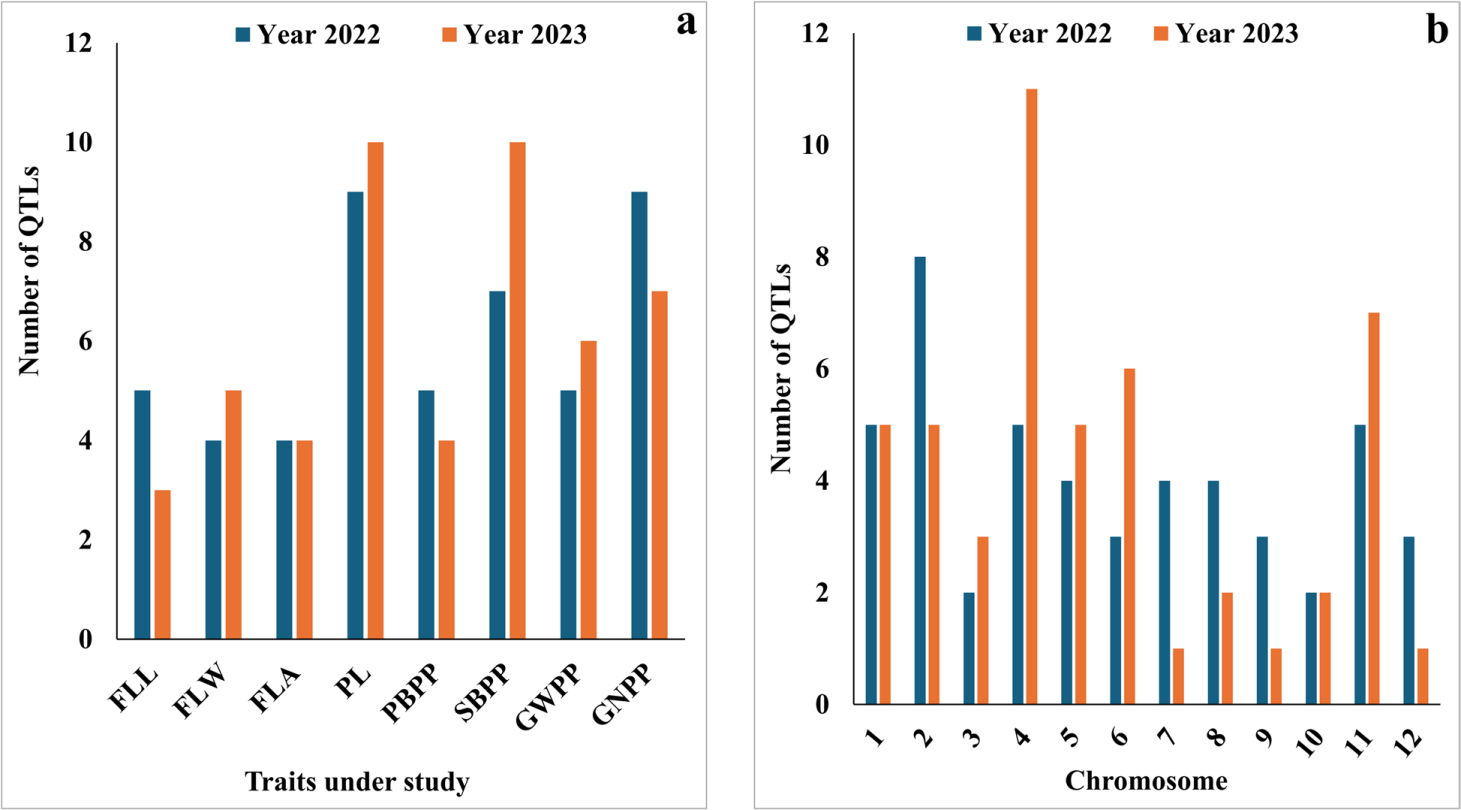
Summary of 97 marker trait associations (MTAs) with flag leaf and panicle architecture: Distribution of MTAs with different traits across the environments (**a**), and Distribution of the MTAs on all 12 chromosomes (**b**)

**Fig. 4:**
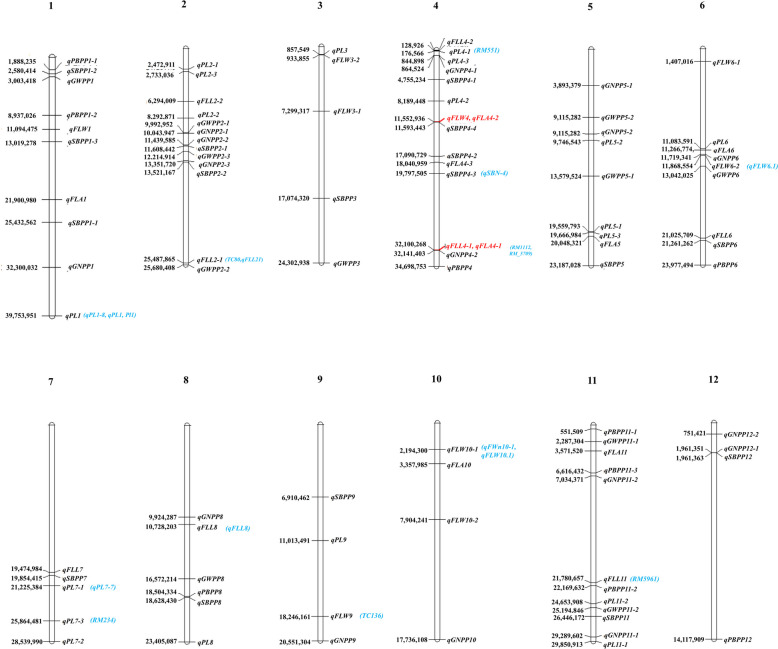
Distribution of quantitative trait loci (QTLs) identified for FLPA-related traits on the rice chromosomes during 2022 and 2023. Each QTL is depicted as a horizontal bar, with the QTL name on the right side and its physical position (bp) on the left side. The QTLs highlighted in red color represent multi-trait QTLs. The light blue color represents the overlapped QTLs with the previous study (for all previous QTL references, see supplementary Table S1)

The GWAS analysis was carried out utilizing 854,832 SNP markers using six multi-locus models independently. Adjusted mean data of each year (2022 and 2023) were used for GWAS analysis for all FLPA-related traits. Based on pooled ANOVA, the years had a significant impact on all traits; hence, GWAS was performed independently for 2022 and 2023 data (Table 1). A total of 97 significant QTLs (Table S1; Fig. 3) were identified for all the studied traits having LOD scores ≥ 3.0 in ≥ 2 multi-locus models. Among them, 48 and 49 QTLs were identified during 2022 and 2023, and 25 and 72 QTLs were identified for FL and panicle-related traits, respectively (Fig. 3). For all eight traits in both years, significant QTLs were shown in Manhattan and quantile-quantile (Q-Q) plots (Fig. S1-S8). PA-related traits were associated with higher number of QTLs than FL traits, with the highest number found in PL (19), followed by SBPP (17). The lowest number of QTLs was found in FLL (8) and FLA (8). The highest number of QTLs were found on chromosome 4 (16 QTLs), followed by 2 (12 QTLs) (Figs. 3 and 4). Four potential QTLs were identified with phenotypic variation (R^2^) ranging from 10.32 to 22.30% (Table 3). Among them, *qGNPP9* (chromosome 9) was identified in 2022, and *qSBPP4-2* (chromosome 4), *qFLW6-2* (chromosome 6), and *qGWPP2-3* (chromosome 2) were identified in 2023.

## Co-localized QTLs for multiple traits

Among all the identified QTLs, two were found as multitrait-QTLs (Table 3). In 2023, the S04_11552936 genomic region on chromosome 4 was linked to two QTLs, *qFLW4* for FLW and *qFLA4-2* for FLA. In 2022, the S04_32100268 genetic locus located on chromosome 4 was linked with two QTLs, namely *qFLL4-1* for FLL and *qFLA4-1* for FLA.

**Fig. 5:**
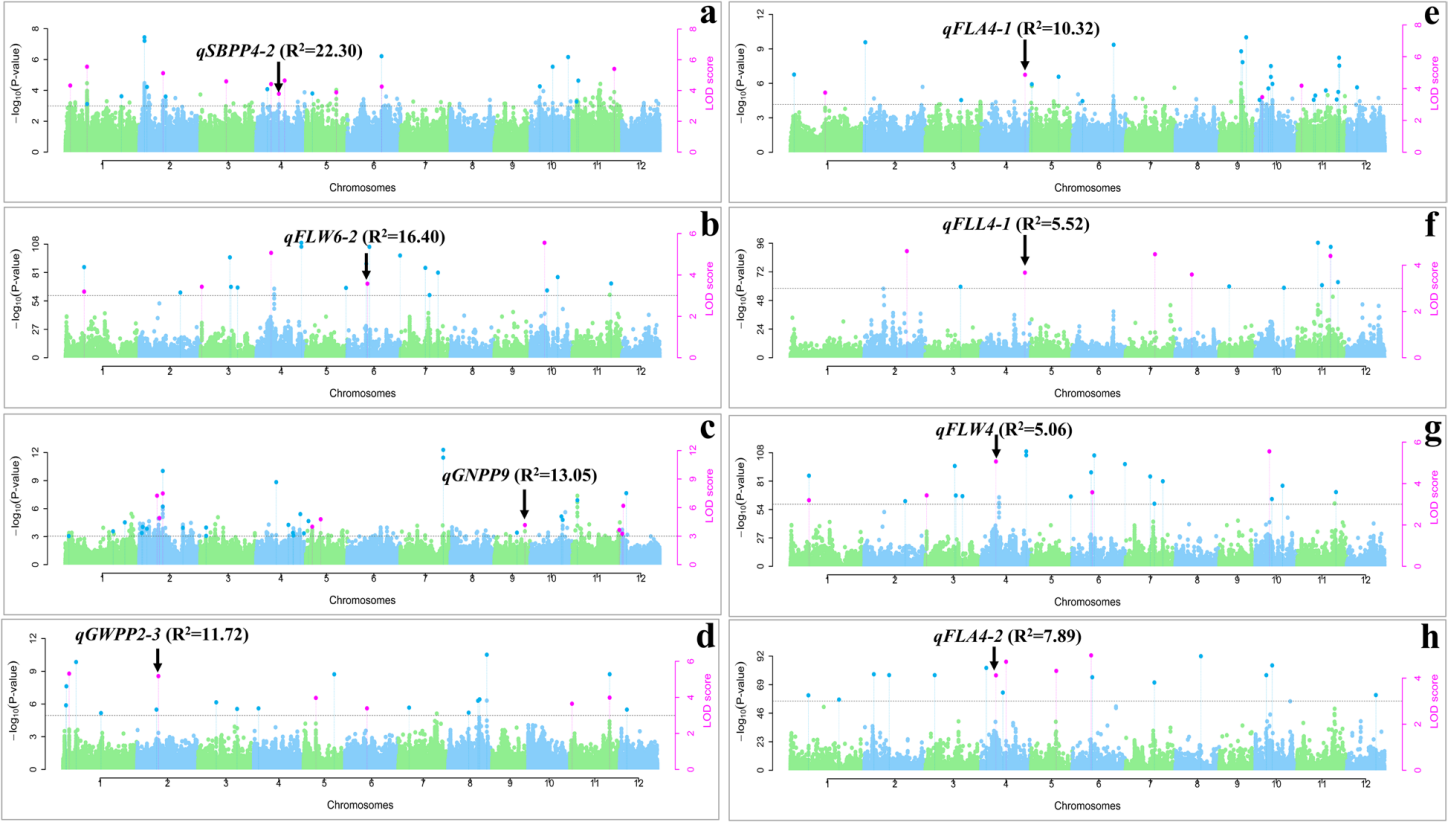
Potential QTLs selected for candidate gene analysis and manhattan plots showing significant SNP-trait associations single trait (**a**-**d**) and co-localized QTLs with two trait (e and f on same genetic loci while g and h on the same genetic loci): *qSBPP4-2* QTL identified for SBPP during 2023 (**a**), *qFLW6-2* QTL identified for FLW during 2023 (**b**), *qGNPP9* QTL identified for GNPP during 2022 (**c**), *qGWPP2-3* QTL identified for GWPP during 2023 (**d**), *qFLA4-1* QTL identified for FLA during 2022 (**e**), *qFLL4-1* QTL identified for FLL during 2022 (**f**), *qFLW4* QTL identified for FLW during 2023 (**g**), and *qFLA4-2* QTL identified for FLA during 2023 (**h**)

Based on specific genomic regions (within 0.5 Mb of a locus region), six genetic loci were associated with the multiple traits (Fig. 5). On chromosome 2, the genomic region from 13.35 to 13.52 Mb was associated with two QTLs: *qGNPP2-3* (for GNPP in 2022) and *qSBPP2-2* (for SBPP in 2023). Similarly, the 0.12–0.17 Mb genomic region on chromosome 4 was associated with *qFLL4-2* (for FLL in 2023) and *qPL4-1* (for PL in 2022), while the 32.10–32.14 Mb region was linked to *qFLA4-1* (for FLA in 2022) and *qGNPP4-2* (for GNPP in 2023). Chromosome 6, 11.06–11.26 Mb region contains two QTLs: *qPL6* (identified in 2022 for PL) and *qFLA6* (identified in 2023 for FLA). QTLs *qPBPP8* (2023 for PBPP) and *qSBPP8* (2022 for SBPP) was located in the 18.50–18.62 Mb interval on chromosome 8. The genomic interval of 21.78–21.22 Mb on chromosome 11 is associated with two QTLs namely *qFLL11* and *qPBPP11-2* for FLL in 2022 and PBPP in 2023, respectively.

### Identification of candidate genes and expression analysis

For candidate gene analysis, among the 97 QTLs, four genetic loci (S04_17090729, S06_11868554, S09_20551304, and S02_12214914) were selected based high phenotypic variation explained (≥ 10), and two genetic loci (S04_11552936 and S04_32100268) were selected based on potential pleotropism (Table 3). Based on the calculated LD decay (150 kb) of the population [49, 52], we took 100 kb upstream and downstream of each marker position as the target region for candidate gene identification. A total of 65 putative candidate genes were found in the target region of the six QTL locations (Table S2), and among them, three genes were associated with the protein kinase domain. Three QTLs associated with PA, such as *qSBPP4-2* on chromosome 4, *qGNPP9* on chromosome 9, and *qGWPP2-3* on chromosome 2, revealed 5, 21, and 4 putative candidate genes, respectively (Table S2). For FL architecture, *qFLW6-2* QTL on chromosome 6 revealed 10 putative candidate genes in the region. Five and twenty-five potential candidate genes for FL-related traits have been identified by two multi-trait genetic loci, namely S04_11552936 associated with *qFLW4* and *qFLA4-2* on chromosome 4 and S04_32100268 linked to *qFLL4-1* and *qFLA4-1* on chromosome 4 (Table S2). The *Os04g0356600* gene, linked to protein kinase and its responsibility for SBPP, has been identified by the *qSBPP4-2* QTL. The heat shock transcription factor family and zinc finger ring type protein are linked to the *Os09g0526600* and *Os09g0525400* gene, which has been found in the region of *qGNPP9* QTL for GNPP on chromosome 9. The fasciclin-like arabinogalactan protein associated with *Os02g0308400* gene was identified in *qGWPP2-3* QTL, which is related to GWPP trait. *Os04g0273100* and *Os04g0276200* genes belong to the transposase protein and the pumilio homology domain protein, respectively. In addition, these genes were found from multi-trait genetic loci (S04_11552936) that are linked to the *qFLW4* and *qFLA4-2* QTLs. S04_32100268 genetic loci associated with *qFLL4-1* and *qFLA4-1* for FLL and FLA, respectively, on chromosome 4 was neighbored by two genes, *Os04g0631100* and *Os04g0631600*. These genes are associated with important proteins like sugar transporter conserved site and transcription factor bHLH95-like basic helix-loop-helix domain, respectively, which help with genetic improvement for FL traits.

**Fig. 6:**
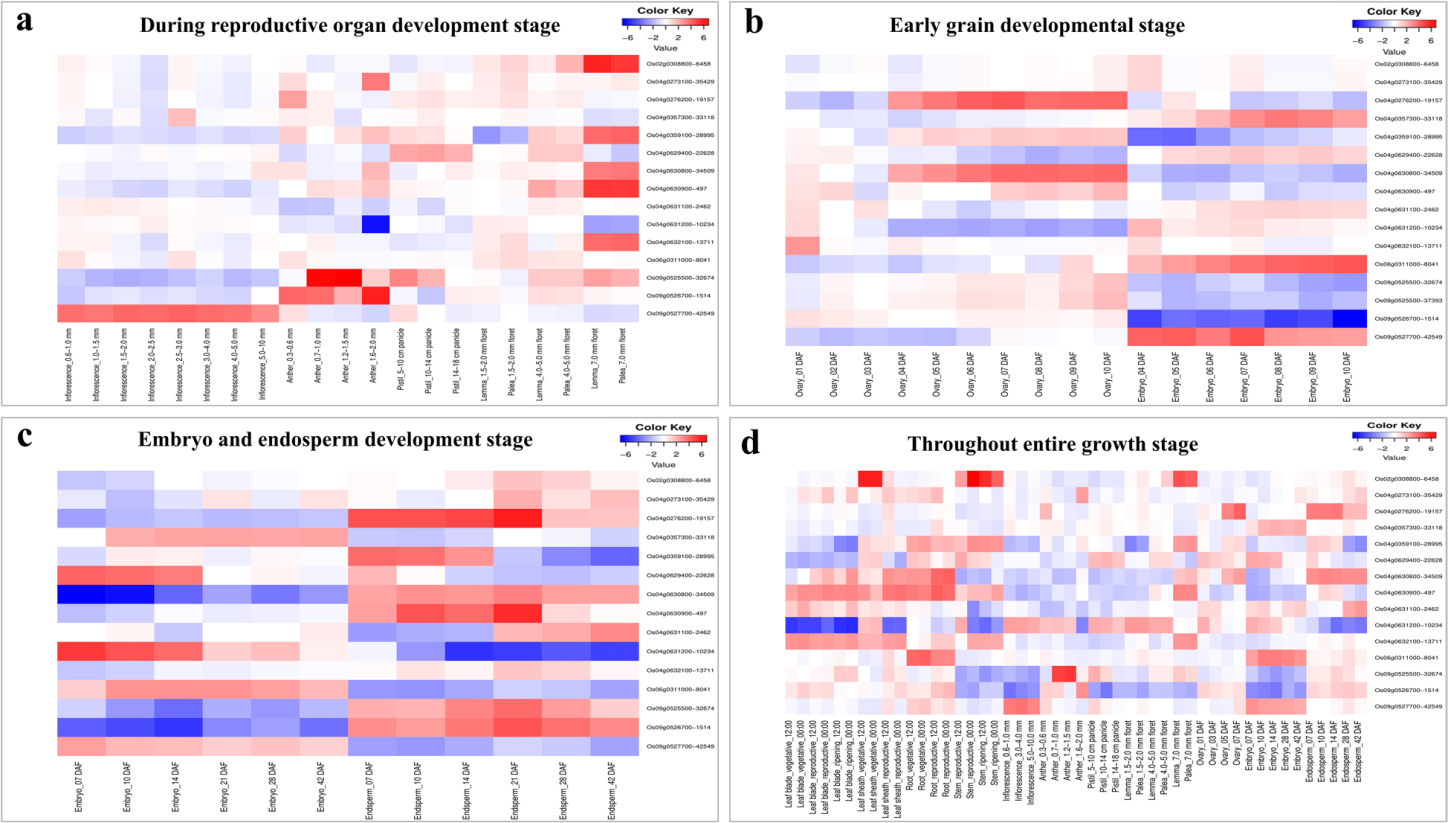
Heatmap for potential putative candidate gene expression analysis for FLPA-related traits through RiceXPro: putative candidate gene expression during reproductive organ development stage (**a**), putative candidate gene expression during early grain developmental stage (**b**), putative candidate gene expression during embryo as well as endosperm development stage, (**c**) and putative candidate gene expression throughout the entire crop growth (**d**). Red represents higher gene expression and green indicates lower gene expression level

RiceXPro tool was used for expression analysis of 65 identified candidate genes (Fig. S9 and S10) for the crop development and reproductive stages (Fig. 6), and potential candidate genes were identified for FLPA-related traits (Table 4). Based on expression levels, three genes were identified, including *Os04g0273100*, *Os04g0631100*, and *Os04g0632100*, which were associated with transposase, sugar transporter, and protein kinase, which is highly expressed in endosperm and palea development stages (Table 4).

**Table 3:** Potential QTLs identified for FLPA-related traits from diverse rice accessions

S. No.	QTL name	Trait name	Year	Chromosome	Marker ID	Marker position (bp)	LOD score	*R*^2^(%)	Method
QTLs identified with major phenotypic effect									
1.	*qSBPP4-2*	SBPP	2023	4	S04_17090729	17,090,729	3.97	22.30	1, 2, 3, 5
2.	*qFLW6-2*	FLW	2023	6	S06_11868554	11,868,554	3.91	16.40	1, 5
3.	*qGNPP9*	GNPP	2022	9	S09_20551304	20,551,304	5.00	13.05	3, 4
4.	*qGWPP2-3*	GWPP	2023	2	S02_12214914	12,214,914	5.69	11.72	1, 2, 3, 4, 5
Multi-trait QTLs (Co-localized QTLs)									
5.	*qFLW4*	FLW	2023	4	S04_11552936	11,552,936	4.86	5.06	2, 5
6.	*qFLA4-2*	FLA	2023	4	S04_11552936	11,552,936	4.89	7.19	2, 5
7.	*qFLL4-1*	FLL	2022	4	S04_32100268	32,100,268	4.48	5.52	1, 2, 5
8.	*qFLA4-1*	FLA	2022	4	S04_32100268	32,100,268	4.93	10.32	2, 5

Model: 1 = FASTmrMLM, 2 = pLARmEB, 3 = mrMLM, 4 = pKWmEB, and 5 = ISIS EM-BLASSO

*FLL *Flag leaf length, *FLW*  Flag leaf width, *FLA*  Flag leaf area, *SBPP*  Secondary branch per panicle, *GWPP*  Grain weight per panicle, and *GNPP* Grain number per panicle.

**Table 4:** List of potential candidate genes associated with FLPA-related traits in rice that are highly expressed during the reproductive stage

QTL name	Marker ID	Chromosome	Gene stable ID	Transcript stable ID	Gene start (bp)	Gene end (bp)	Protein description
*qSBPP4-2*	S04_17090729	4	*Os04g0356600*	Os04t0356600-01	17,044,349	17,046,921	Protein kinase domain
			*Os04g0357300*	Os04t0357300-01	17,063,617	17,065,887	Similar to helicase-like protein
			*Os04g0359100*	Os04t0359100-01	17,180,207	17,183,428	Polyketide synthase, enoylreductase domain
*qFLW6-2*	S06_11868554	6	*Os06g0311000*	Os06t0311000-00	11,840,611	11,844,171	Glycosyltransferase 61
*qGNPP9*	S09_20551304	9	*Os09g0525400*	Os09t0525400-01	20,528,648	20,532,222	Zinc finger, RING-type
			*Os09g0526700*	Os09t0526700-02	20,600,616	20,603,003	UDP-glucose 4-epimerase
			*Os09g0526600*	Os09t0526600-02	20,591,252	20,595,143	Heat shock transcription factor family
			*Os09g0525500*	Os09t0525500-01	20,534,165	20,534,903	Bifunctional inhibitor/plant lipid transfer protein/seed storage helical domain
			*Os09g0527700*	Os09t0527700-01	20,641,465	20,643,139	AUX/IAA protein
*qGWPP2-3*	S02_12214914	2	*Os02g0308400*	Os02t0308400-02	12,113,907	12,114,966	Fasciclin-like arabinogalactan protein, group A
			*Os02g0308800*	Os02t0308800-01	12,127,913	12,129,233	FAS1 domain
*qFLW4* and *qFLA4-2*	S04_11552936	4	*Os04g0273100*	Os04t0273100-01	11,463,290	11,464,387	Similar to transposase
			*Os04g0276200*	Os04t0276200-01	11,650,394	11,660,149	Pumilio homology domain
*qFLL4-1* and *qFLA4-1*	S04_32100268	4	*Os04g0629400*	Os04t0629400-01	32,017,656	32,018,383	Uncharacterized protein Os04g0629400-like
			*Os04g0630800*	Os04t0630800-01	32,085,612	32,088,272	Similar to anthocyanidin reductase
			*Os04g0630900*	Os04t0630900-01	32,090,880	32,093,039	Similar to H0105C05.3 protein
			*Os04g0631100*	Os04t0631100-01	32,139,084	32,140,993	Sugar transporter, conserved site
			*Os04g0631200*	Os04t0631200-02	32,145,681	32,147,446	Glycoside hydrolase family 16
			*Os04g0632100*	Os04t0632100-01	32,178,831	32,182,153	Protein kinase, ATP binding site

## Discussion

Understanding the genetic basis for FLPA is a critical step in developing molecular markers to enhance breeding efficiency and rice grain yield. Rice FL is the primary photosynthetic organ during grain filling, and its structural characteristics directly impact light interception, light dispersion, and energy usage. As a result, it is a major target trait for rice breeding programs to improve grain yield [68]. According to reports, FL photosynthesis supplies more than half of the carbohydrates in rice seeds [10]. The FLA is a critical factor in determining photosynthetic capability; it has two primary components, i.e., FLL and FLW, and is regulated by many QTL and their interactions with the environment. Similarly, PA-related traits (PL, PBPP, SBPP, GWPP, and GNPP) are complex quantitative traits regulated by multiple genes and controlled by the environment [14, 15, 69]. Both FL and PA-related traits are important components of improving rice yield. The diversity of genetic variation in rice germplasm accessions is an important and potential source of significant genetic loci. Few reports [14, 70–73] were published earlier on GWAY analysis using FLPA traits, however study was done using new germplasm phenotyped twice in a US environment to explore further and expand knowledge regarding the trait diversity. To further understand the genetic basis of FLPA and its importance in rice breeding, dissecting the QTL regulated by unique genomic locations and those important genomic region experess under various environmental factors is necessary. Our findings will help in the design of molecular breeding techniques for developing new rice cultivars as well as finding germplasms suitable for rice breeding and improvement.

### Phenotypic variations for targeted traits

GWAS analysis results depend on the precise and appropriate phenotyping of the target traits. This study looked at two broad categories: FL and PA-related traits in two distinct environments. During 2022 and 2023, 208 diverse germplasm accessions’ data for all parameters showed normal distributions (Fig. 1), which was appropriate for GWAS analysis. We performed a Shapiro-Wilk test for normality, and it was determined that all study traits almost equal to normal distribution. Previously, the same population was used for a GWAS on key agronomic traits and quality parameters using data from 2018 to 2019 [49, 52]. Significant variability among the genotypes found in this study in both years for most studied traits was supported by previously published research [14, 74]. The mean value of all FLPA-related attributes decreased in 2022 compared to 2023 (Table 2), indicating that genetic and environment-related factors influenced these quantitative traits. The yield of rice grain is considered as an important polygenic trait influenced by many phenotypic traits, including FLPA, along with many environmental factors [75, 76]. FL-related (FLL, FLW, and FLA) traits CV ranged from 6.13 to 14.73 (Table 1), which was similar to the previous study [77]. For the PA (PL, PBPP, SBPP, GWPP, and GNPP) trait, CV ranged 6.72–36.74 (Table 1), which were higher than FL traits, these results were supported by earlier studies [70, 75, 78]. These results indicated that, being a quantitative nature of FLPA traits, FL was less affected by environmental factors compared to PA during both years. Most of the traits under study showed a positive correlation except SBPP (Fig. 2). For FL-realted traits, FLA showed a positive and significant correlation (r = > 0.7) with FLL and FLW in both years (Fig. 3). Panicle archititure traits, GNPP showed a positive correlation (between *r* = 0.2–0.6) with all traits except SBPP during 2022, 2023, and the mean of both years. These association analysis indicators effectively reflect the FL compared to panicle traits, which are beneficial for improving rice grain yield by GWAS, our results were also supported by earlier studies [72, 73]. Overall, phenotypic data revealed that several significant genetic regions may be responsible for FLPA-related attributes, and the researcher needs to further investigate distinct germplasm accession for GWAS analysis before reaching a final conclusion.

### Genome-wide association study for FLPA-related traits

The speedy advancement of genome sequencing technology has made GWAS an effective tool for QTL analysis and has been utilized widely in rice research for analyzing complex features, including FLPA [12, 71, 73]. GWAS is a groundwork study that identifies genomic regions associated with FLPA-related traits. Using 854,832 polymorphic SNPs [49, 52] for this investigation, we were able to identify 97 QTLs (48 in 2022 and 49 in 2023) (Fig. S1-S8) out of which 24 were found based on > 3 models and were all significantly associated with all studied traits (Table S1). Previously, a GWAS study used a diverse set of rice germplasm accessions to identify FLPA related traits and revealed eight QTLs [79], 15 QTLs [73], and 64 QTLs [12] for FL-related traits, as well as 29 QTLs [40], 153 QTLs [14], 106 QTLs [71], and 49 QTLs [72] for PA-related traits. During 2022 and 2023, the present study found 25 and 72 QTLs for FL and PA-related traits, respectively (Fig. 4 and Table S1). Earlier research focused on FL and PA-related traits individually, but our objective was to combine both traits in a GWAS to discover suitable markers that would aid in developing rice cultivars with enhanced FLPA-related traits.

### Comparison of identified QTLs with previously published FLPA genes/QTLs

Multiple studies explored diverse rice germplasms for QTLs related to FLPA traits. As a result, reviewing published literature to identify overlapped QTLs will validate the importance of specific genomic regions. From this study, 12 QTLs overlapped with previously reported QTLs. Four QTLs related to FLL were reported earlier (Fig. 4 and Table S1). Several of our QTLs overlap with previously reported QTLs/genes, revealing consistent genetic regions influencing these traits. For example, *qFLL2-1* coincided with previously identified QTLs *TC80* [68] and *qFLL2.1* [80]. Another QTL *qFLL4-1* is linked to the single sequence repeat (SSR) markers *RM1112* [68] and *RM_5709* [81], which were previously reported to be significantly associated with FLL. Further, *qFLL8* identified from our study was also reported previously [31]. Three QTLs linked with FLW were also documented earlier. For example, *qFLW9* identified in this study overlapped with *TC136* [68], *qFLW10-1* was reported as *qFWn10-1* [12], and *qFLW10.1* [80] in previous studies. These results will help narrow down the identified QTLs for fine-mapping FL-related traits. Likewise, some QTLs controlling PA were also reported earlier (Fig. 4 and Table S1). For example, *qSBPP4-3* QTL controlling SBPP overlapped with *qSBN-4* [72]. Also, four QTLs related to PL were documented previously in multiple studies. For example, *qPL1* identified in our study overlapped with *qPL1-8* [14], *qPL1* [82], and *qPl1* [13], *qPL4-1* overlapped with *RM551* [81], *qPL7-1* and *qPL7-3* overlapped with *qPL7-7* [14] and *RM_234* [81], respectively. Future research should focus on comparing identified QTLs with overlapping QTLs to refine genetic markers, discover new functional mechanisms, and integrate omics data. This will improve trait understanding, boost breeding efficiency, and expand applications across diverse rice germplasms.

### Putative candidate genes and expression analysis

To further refine the potential identification of QTLs, six specific genetic loci were selected for candidate gene analysis (Table 3; Fig. 5). This selection aims to narrow down the potential candidate genes associated with the QTLs by focusing on these loci, allowing for a more precise investigation into their roles and effects on the traits of interest. With a resolution of 100 kb radius genomic regions at the identified QTL locations, a minimal number of possible genes were identified; a similar approach was also utilized to identify the candidate genes in previously published research [83–85]. Of the 97 QTLs, four were chosen based on phenotypic variation (> 10%) and two for their pleiotropic effects on multiple traits for candidate gene analysis (Table 3). A total of 65 potential putative candidate genes were identified from Ensemble plants (Table S2). We identified 35 candidate genes for FL and 30 for PA traits. These putative QTLs give the foundation information for future studies to identify the FLPA-related genomic region from diverse rice germplasm.

Protein kinase plays an essential role during panicle development, especially panicle apical spikelets [24]. In the present study, one of the SBPP trait controlling the QTLs, *qSBPP4-2*, which is identified as *Os04g0356600* gene and is associated with protein kinases. The protein heat shock transcription factor family has the highest levels of gene expression in reproductive organs, where it regulates the embryo and lemma to enhance rice grain yield [25]. Similarly, this protein has been linked to spikelet fertility in rice [86] and anther development in wheat [87]. Zinc-finger ring-type protein expressed during the spikelet development in rice [88]. From *qGNPP9* QTL, we identified the *Os09g0526600* and *Os09g0525400* genes, which are linked to the heat shock transcription factor family and zinc finger ring type protein, respectively. The sugar transport protein family plays an important role in sink tissue (the major site of carbohydrate storage in leaves), which helps with grain filling in rice [26] and different stages of plant development [27, 28]. Our study also found the *Os04g0631100* gene for *qFLL4-1* and *qFLA4-1* QTLs, which are associated with the sugar transporter protein. In rice, the bHLH (basic helix-loop-helix) protein family influences FL shape and angle [29, 79]. For *qFLL4-1* and *qFLA4-1* QTLs, our study identified the *Os04g0631600* gene that corresponds to the same protein family and directly contributes to improving FL traits.

Plant molecular biology uses several strategies that are improved by gene expression profiling, especially when characterizing gene function [89]. In the current study, we used the gene expression profile database, i.e., RiceXPro (https://ricexpro.dna.affrc.go.jp/index.html), to find more comprehensive information on the rice transcriptome, encompassing the entire growth cycle as well as the reproductive stage at field experimental conditions [45, 67, 89]. A total of 65 potential putative candidate genes were identified in this study (Table S2; Fig. S9 and S10) throughout the entire crop growth period of crop. Among them, 15 candidate genes (Fig. 6) were highly expressed during the reproductive stage (Table 4). *Os04g0631100* gene was highly expressed during the endosperm development stage and was also associated with sugar transporter protein; further, this gene needs to be validated for functional characterization. Many of the previous studies used RiceXPro to find the potential candidate gene for different traits like reproductive traits [48], roots-related traits [47], and important agronomic traits [46]. In silico expression analysis was chosen for validating gene expression during the reproductive stage because it efficiently screens large datasets to identify candidate genes at this stage. All things considered, the QTLs and potential candidate genes identified in this research suggest that several unexplored factors could play a role in determining FLPA-related phenotypes and be helpful in molecular marker development and marker-assisted selection in rice breeding programs.

## Conclusion

Based on independent GWAS analysis from both years, we identified 97 QTLs with significant associations with all studied traits. Among these, 24 QTLs were found significant in at least three multi-locus models and four of which (*qSBPP4-2*, *qFLW6-2*, *qGNPP9*, and *qGWPP2-3*) showed high phenotypic variance explained ranging from 11.72 to 22.30%, and two genetic loci were linked to multi-trait QTLs. Overall, 12 QTLs were found to be reported in previous studies for FLPA traits, and among them, seven QTLs were associated with FL-related traits, and five QTLs with PA. Two multi-trait QTLs, S04_32100268 (*qFLL4-1* and *qFLA4-1*) and S04_11552936 (*qFLW4* and *qFLA4-2*) were found associated with 65 putative candidate genes. In silico expression analysis by RiceXPro revealed that 15 candidate genes were highly expressed during the reproductive stage. This approach is essential for FLPA traits, as reproductive stage-specific expression significantly affects fertility and contributes to increased grain yield, thus providing important insights for rice breeding strategies. Among 15 candidate genes, five putative candidate genes i.e., *Os04g0356600*, *Os09g0526600*, *Os09g0525400*, *Os04g0631100*, and *Os04g0631600*, were associated with protein kinase, heat shock transcription factor family, zinc-finger ring-type, sugar transporter conserved site, and transcription factor bHLH95-like basic helix-loop-helix domain, respectively, regulating different FLPA-realted traits. These genes will be the focus for future research into their possible roles in the rice breeding program.

## Supplementary Information

Supplementary Material 1: Table S1. Summary of 97 QTLs associated with flag leaf and panicle architecture-related traits during 2022 and 2023.

Supplementary Material 2: Table S2. Summary of 65 putative candidate genes located in the six potential genetic loci intervals.

Supplementary Material 3: Fig. S1. Manhattan and quantile-quantile (Q-Q) plots showing significant marker trait associations with flag leaf length: During 2022 (a), and during 2023 (b).

Supplementary Material 4: Fig. S2. Manhattan and quantile-quantile (Q-Q) plots showing significant marker trait associations with flag leaf width: During 2022 (a), and during 2023 (b).

Supplementary Material 5: Fig. S3. Manhattan and quantile-quantile (Q-Q) plots showing significant marker trait associations with flag leaf area: During 2022 (a), and during 2023 (b).

Supplementary Material 6: Fig. S4. Manhattan and quantile-quantile (Q-Q) plots showing significant marker trait associations with panicle length: During 2022 (a), and during 2023 (b).

Supplementary Material 7: Fig. S5. Manhattan and quantile-quantile (Q-Q) plots showing significant marker trait associations with primary branch per panicle: During 2022 (a), and during 2023 (b).

Supplementary Material 8: Fig. S6. Manhattan and quantile-quantile (Q-Q) plots showing significant marker trait associations with secondary branch per panicle: During 2022 (a), and during 2023 (b).

Supplementary Material 9: Fig. S7. Manhattan and quantile-quantile (Q-Q) plots showing significant marker trait associations with grain weight per panicle: During 2022 (a), and during 2023 (b).

Supplementary Material 10: Fig. S8. Manhattan and quantile-quantile (Q-Q) plots showing significant marker trait associations with grain number per panicle: During 2022 (a), and during 2023 (b).

Supplementary Material 11: Fig. S9. All QTLs name should be in italic format likeq *SBPP4-2*, *qFLW6-2*, *qGNPP9*, and *GWPP2-3*.

Supplementary Material 12: Fig. S10. All QTLs name should be in italic format likeq *FLW4*, *qFLA4-2*, *qFLL4-1* and *qFLA4-1*.

## Data Availability

The datasets generated and/or analyzed during the current study are not publicly available due to sensitivity but are available from the authors on reasonable request.

## References

[1] Prasad R, Shivay YS, Kumar D. Current status, challenges, and opportunities in rice production. In: Rice production worldwide. 2017. pp. 1–32.

[2] Avinash G, Sharma N, Prasad KR, Kaur R, Singh G, Pagidipala N, et al. Unveiling the distribution of free and bound phenolic acids, flavonoids, anthocyanins, and proanthocyanidins in pigmented and non-pigmented rice genotypes. Front Plant Sci. 2024;15:1324825. 38660452 10.3389/fpls.2024.1324825 PMC11039891

[3] Kaur R, Kaur R, Sharma N, Kumari N, Khanna R, Singh G. Protein profiling in a set of wild rice species and rice cultivars: a stepping stone to protein quality improvement. Cereal Res Commun. 2023;51:163–77.

[4] Fahad S, Adnan M, Noor M, Arif M, Alam M, Khan IA et al. Major constraints for global rice production. In: Advances in rice research for abiotic stress tolerance. 2019. pp. 1–22.

[5] Jyoti SD, Singh G, Pradhan AK, Tarpley L, Septiningsih EM, Talukder SK. Rice breeding for low input agriculture. Front Plant Sci. 2024;15:1408356. 38974981 10.3389/fpls.2024.1408356 PMC11224470

[6] Nawaz A, Rehman AU, Rehman A, Ahmad S, Siddique KH, Farooq M. Increasing sustainability for rice production systems. J Cereal Sci. 2022;103:103400.

[7] Spindel JE, Begum H, Akdemir D, Collard B, Redoña E, Jannink JL, et al. Genome-wide prediction models that incorporate de novo GWAS are a powerful new tool for tropical rice improvement. Heredity (Edinb). 2016;116:395–408. 26860200 10.1038/hdy.2015.113 PMC4806696

[8] Xing Y, Zhang Q. Genetic and molecular bases of rice yield. Annu Rev Plant Biol. 2010;61:421–42. 20192739 10.1146/annurev-arplant-042809-112209

[9] Yuan S, Li Y, Peng S. Leaf lateral asymmetry in morphological and physiological traits of rice plant. PLoS ONE. 2015;10:e0129832. 26053267 10.1371/journal.pone.0129832 PMC4460030

[10] Li Z, Pinson SR, Stansel JW, Paterson AH. Genetic dissection of the source-sink relationship affecting fecundity and yield in rice (*Oryza sativa* L). Mol Breed. 1998;4:419–26.

[11] Wang P, Zhou G, Yu H, Yu S. Fine mapping a major QTL for flag leaf size and yield-related traits in rice. Theor Appl Genet. 2011;123:1319–30. 21830109 10.1007/s00122-011-1669-6

[12] Wang J, Wang T, Wang Q, Tang X, Ren Y, Zheng H et al. QTL mapping and candidate gene mining of flag leaf size traits in Japonica rice based on linkage mapping and genome-wide association study. Mol Biol Rep. 2022;:1–9. 10.1007/s11033-021-06842-8 34677716

[13] Lim JH, Yang HJ, Jung KH, Yoo SC, Paek NC. Quantitative trait locus mapping and candidate gene analysis for plant architecture traits using whole genome re-sequencing in rice. Mol Cells. 2014;37:149–60. 24599000 10.14348/molcells.2014.2336 PMC3935628

[14] Bai S, Hong J, Li L, Su S, Li Z, Wang W, et al. Dissection of the genetic basis of rice panicle architecture using a genome-wide association study. Rice. 2021;14:1–12. 34487253 10.1186/s12284-021-00520-w PMC8421479

[15] Singh G, Kaur N, Khanna R, Kaur R, Gudi S, Kaur R, et al. 2Gs and plant architecture: breaking grain yield ceiling through breeding approaches for next wave of revolution in rice (*Oryza sativa* L). Crit Rev Biotechnol. 2024;44:139–62. 36176065 10.1080/07388551.2022.2112648

[16] Bai X, Zhao H, Huang Y, Xie W, Han Z, Zhang B, et al. Genome-wide association analysis reveals different genetic control in panicle architecture between Indica and Japonica rice. Plant Genome. 2016;9:1–10. 10.3835/plantgenome2015.11.0115 27898816

[17] Li S, Zhao B, Yuan D, Duan M, Qian Q, Tang L, et al. Rice zinc finger protein *DST* enhances grain production through controlling *Gn1a*/OsCKX2 expression. Proc Natl Acad Sci. 2013;110:3167–72. 23382237 10.1073/pnas.1300359110 PMC3581943

[18] Sun P, Zhang W, Wang Y, He Q, Shu F, Liu H, et al. *OsGRF4* controls grain shape, panicle length and seed shattering in rice. J Integr Plant Biol. 2016;58:836–47. 26936408 10.1111/jipb.12473 PMC5089622

[19] Zhu Z, Tan L, Fu Y, Liu F, Cai H, Xie D, et al. Genetic control of inflorescence architecture during rice domestication. Nat Commun. 2013;4:2200. 23884108 10.1038/ncomms3200 PMC3731664

[20] Yu H, Qiu Z, Xu Q, Wang Z, Zeng D, Hu J, et al. Fine mapping of LOW TILLER 1, a gene controlling tillering and panicle branching in rice. Plant Growth Regul. 2017;83:93–104.

[21] Miura K, Ikeda M, Matsubara A, Song XJ, Ito M, Asano K, et al. OsSPL14 promotes panicle branching and higher grain productivity in rice. Nat Genet. 2010;42:545–9. 20495564 10.1038/ng.592

[22] Song X, Meng X, Guo H, Cheng Q, Jing Y, Chen M, et al. Targeting a gene regulatory element enhances rice grain yield by decoupling panicle number and size. Nat Biotechnol. 2022;40:1403–11. 35449414 10.1038/s41587-022-01281-7

[23] Li G, Tang J, Zheng J, Chu C. Exploration of rice yield potential: decoding agronomic and physiological traits. Crop J. 2021;9:577–89.

[24] Peng Y, Hou F, Bai Q, Xu P, Liao Y, Zhang H, et al. Rice calcineurin B-like protein-interacting protein kinase 31 (OsCIPK31) is involved in the development of panicle apical spikelets. Front Plant Sci. 2018;9:1661. 30524455 10.3389/fpls.2018.01661 PMC6262370

[25] Shamshad A, Rashid M, Zaman QU. In-silico analysis of heat shock transcription factor (OsHSF) gene family in rice (*Oryza sativa* L). BMC Plant Biol. 2023;23:395. 37592226 10.1186/s12870-023-04399-1 PMC10433574

[26] Takeda T, Toyofuku K, Matsukura C, Yamaguchi J. Sugar transporters involved in flowering and grain development of rice. J Plant Physiol. 2011;158:465–70.

[27] Aoki N, Hirose T, Scofield GN, Whitfeld PR, Furbank RT. The sucrose transporter gene family in rice. Plant Cell Physiol. 2003;44:223–32. 12668768 10.1093/pcp/pcg030

[28] Wu Y, Lee SK, Yoo Y, Wei J, Kwon SY, Lee SW, et al. Rice transcription factor OsDOF11 modulates sugar transport by promoting expression of sucrose transporter and SWEET genes. Mol Plant. 2018;11:833–45. 29656028 10.1016/j.molp.2018.04.002

[29] Tian Q, Luan J, Guo C, Shi X, Deng P, Zhou Z, et al. A bHLH protein, OsBIM1, positively regulates rice leaf angle by promoting brassinosteroid signaling. Biochem Biophys Res Commun. 2021;578:129–35. 34562652 10.1016/j.bbrc.2021.09.035

[30] Zhu J, Zhou Y, Liu Y, Wang Z, Tang Z, Yi C, et al. Fine mapping of a major QTL controlling panicle number in rice. Mol Breed. 2011;27:171–80.

[31] Zhang B, Ye W, Ren D, Tian P, Peng Y, Gao Y, et al. Genetic analysis of flag leaf size and candidate genes determination of a major QTL for flag leaf width in rice. Rice. 2015;8:1–10. 26054240 10.1186/s12284-014-0039-9 PMC4883130

[32] Okada S, Sasaki M, Yamasaki M. A novel rice QTL *qOPW11* associated with panicle weight affects panicle and plant architecture. Rice. 2018;11:1–9. 30225538 10.1186/s12284-018-0246-x PMC6141410

[33] Sasaki K, Fujita D, Koide Y, Lumanglas PD, Gannaban RB, Tagle AG, et al. Fine mapping of a quantitative trait locus for spikelet number per panicle in a new plant type rice and evaluation of a near-isogenic line for grain productivity. J Exp Bot. 2017;68:2693–702. 28582550 10.1093/jxb/erx128 PMC5853308

[34] Wang SS, Chen RK, Chen KY, Liu CY, Kao SM, Chung CL. Genetic mapping of the *qSBN7* locus, a QTL controlling secondary branch number per panicle in rice. Breed Sci. 2017;67:340–7. 29085243 10.1270/jsbbs.17007 PMC5654460

[35] Kaur A, Sidana K, Bhatia D, Neelam K, Singh G, Sahi GK, et al. A novel QTL *qSPP2. 2* controlling spikelet per panicle identified from *Oryza longistaminata* (A. Chev. Et roehr.), mapped and transferred to Oryza sativa (L). Mol Breed. 2018;38:1–13.

[36] Jiang S, Zhang X, Wang J, Chen W, Xu Z. Fine mapping of the quantitative trait locus *qFLL9* controlling flag leaf length in rice. Euphytica. 2010;176:341–7.

[37] Shen B, Yu WD, Zhu YJ, Fan YY, Zhuang JY. Fine mapping of a major quantitative trait locus, qFLL6. 2, controlling flag leaf length and yield traits in rice (*Oryza sativa* L). Euphytica. 2012;184:57–64.

[38] Bian J, He H, Shi H, Zhu G, Li C, Zhu C, et al. Quantitative trait loci mapping for flag leaf traits in rice using a chromosome segment substitution line population. Plant Breed. 2014;133:203–9.

[39] Tibbs Cortes L, Zhang Z, Yu J. Status and prospects of genome-wide association studies in plants. Plant Genome. 2021;14:e20077. 33442955 10.1002/tpg2.20077 PMC12806871

[40] Ta KN, Khong NG, Ha TL, Nguyen DT, Mai DC, Hoang TG, et al. A genome-wide association study using a Vietnamese landrace panel of rice *(Oryza sativa*) reveals new QTLs controlling panicle morphological traits. BMC Plant Biol. 2018;18:1–15. 30428844 10.1186/s12870-018-1504-1 PMC6234598

[41] Ya-fang Z, Yu-yin MA, Zong-xiang C, Jie ZOU, Tian-xiao C, Qian-qian LI, et al. Genome-wide association studies reveal new genetic targets for five panicle traits of international rice varieties. Rice Sci. 2015;22:217–26.

[42] Liu E, Liu Y, Wu G, Zeng S, Tran Thi TG, Liang L, et al. Identification of a candidate gene for panicle length in rice (*Oryza sativa* L.) via association and linkage analysis. Front Plant Sci. 2016;7:596. 27200064 10.3389/fpls.2016.00596 PMC4853638

[43] Chen S, Liu F, Wu W, Jiang Y, Zhan K. A SNP-based GWAS and functional haplotype-based GWAS of flag leaf-related traits and their influence on the yield of bread wheat (*Triticum aestivum* L). Theor Appl Genet. 2021;134:3895–909. 34436627 10.1007/s00122-021-03935-7

[44] Eizenga GC, Jackson AK, Edwards JD. Prototype for developing SNP markers from GWAS and biparental QTL for rice panicle and grain traits. Agric Environ Lett. 2021;6:e20047.

[45] Sato Y, Antonio BA, Namiki N, Takehisa H, Minami H, Kamatsuki K, et al. RiceXPro: a platform for monitoring gene expression in japonica rice grown under natural field conditions. Nucleic Acids Res. 2010;39:D1141–8. 21045061 10.1093/nar/gkq1085 PMC3013682

[46] Hori K, Shenton M. Current advances and future prospects for Molecular Research for Agronomically important traits in Rice. Int J Mol Sci. 2022;23:7531. 35886876 10.3390/ijms23147531 PMC9316905

[47] Mounier T, Navarro-Sanz S, Bureau C, Antoine L, Varoquaux F, Durandet F, et al. A fast, efficient and high-throughput procedure involving laser microdissection and RT droplet digital PCR for tissue-specific expression profiling of rice roots. BMC Mol Cell Biol. 2020;21:1–17. 33302866 10.1186/s12860-020-00312-y PMC7727186

[48] Akasaka M, Taniguchi Y, Oshima M, Abe K, Tabei Y, Tanaka J. Development of transgenic male-sterile rice by using anther-specific promoters identified by comprehensive screening of the gene expression profile database ‘RiceXPro’. Breed Sci. 2018;68:420–31. 30369816 10.1270/jsbbs.18019 PMC6198903

[49] Sanchez DL, Samonte SOP, Alpuerto JBB, Croaker PA, Morales KY, Yang Y, et al. Phenotypic variation and genome-wide association studies of main culm panicle node number, maximum node production rate, and degree-days to heading in rice. BMC Genomics. 2022;23:390. 35606708 10.1186/s12864-022-08629-y PMC9125873

[50] Aravind J, Wankhede DP, Kaur V, Aravind MJ. Package ‘augmentedRCBD.’ 2021.

[51] R Core Team R. R: A language and environment for statistical computing. 2013.

[52] Sanchez DL, Samonte SOP, Wilson LT. Genetic architecture of head rice and rice chalky grain percentages using genome-wide association studies. Front Plant Sci. 2023;14:1274823. 38046607 10.3389/fpls.2023.1274823 PMC10691675

[53] Browning SR, Browning BL. Rapid and accurate haplotype phasing and missing-data inference for whole-genome association studies by use of localized haplotype clustering. Am J Hum Genet. 2007;81:1084–97. 17924348 10.1086/521987 PMC2265661

[54] Bradbury PJ, Zhang Z, Kroon DE, Casstevens TM, Ramdoss Y, Buckler ES. TASSEL: software for association mapping of complex traits in diverse samples. Bioinformatics. 2007;23:2633–5. 17586829 10.1093/bioinformatics/btm308

[55] Pritchard JK, Stephens M, Donnelly P. Inference of population structure using multilocus genotype data. Genetics. 2000;155:945–59. 10835412 10.1093/genetics/155.2.945 PMC1461096

[56] VanRaden PM. Efficient methods to compute genomic predictions. J Dairy Sci. 2008;91:4414–23. 18946147 10.3168/jds.2007-0980

[57] Alpuerto JBB, Samonte SOP, Sanchez DL, Croaker PA, Wang YJ, Wilson LT, et al. Genomic association mapping of apparent amylose and protein concentration in milled rice. Agronomy. 2022;12:857.

[58] Wen Y, Zhang Y, Zhang J, Feng J, Zhang Y. The improved FASTmrEMMA and GCIM algorithms for genome-wide association and linkage studies in large mapping populations. Crop J. 2020;8:723–32.

[59] Gudi S, Saini DK, Halladakeri P, Singh G, Singh S, Kaur S, et al. Genome-wide association study unravels genomic regions associated with chlorophyll fluorescence parameters in wheat (*Triticum aestivum* L.) under different sowing conditions. Plant Cell Rep. 2023;42:1453–72. 37338572 10.1007/s00299-023-03041-6

[60] Gudi S, Halladakeri P, Singh G, Kumar P, Singh S, Alwutayd KM, et al. Deciphering the genetic landscape of seedling drought stress tolerance in wheat (*Triticum aestivum* L.) through genome-wide association studies. Front Plant Sci. 2024;15:1351075. 38510445 10.3389/fpls.2024.1351075 PMC10952099

[61] Tamba CL, Zhang YM. A fast mrMLM algorithm for multi-locus genome-wide association studies. Biorxiv. 2018;:341784.

[62] Wen YJ, Zhang H, Ni YL, Huang B, Zhang J, Feng JY, et al. Methodological implementation of mixed linear models in multi-locus genome-wide association studies. Brief Bioinform. 2018;19:700–12. 28158525 10.1093/bib/bbw145 PMC6054291

[63] Ren WL, Wen YJ, Dunwell JM, Zhang YM. pKWmEB: integration of Kruskal–Wallis test with empirical Bayes under polygenic background control for multi-locus genome-wide association study. Heredity (Edinb). 2018;120:208–18. 29234158 10.1038/s41437-017-0007-4 PMC5836593

[64] Zhang J, Feng JY, Ni YL, Wen YJ, Niu Y, Tamba CL, et al. pLARmEB: integration of least angle regression with empirical Bayes for multilocus genome-wide association studies. Heredity (Edinb). 2017;118:517–24. 28295030 10.1038/hdy.2017.8 PMC5436030

[65] Tamba CL, Ni YL, Zhang YM. Iterative sure independence screening EM-Bayesian LASSO algorithm for multi-locus genome-wide association studies. PLoS Comput Biol. 2017;13:e1005357. 28141824 10.1371/journal.pcbi.1005357 PMC5308866

[66] Zhao Z, Zhang Z, Ding Z, Meng H, Shen R, Tang H, et al. Public-transcriptome-database-assisted selection and validation of reliable reference genes for qRT-PCR in rice. Sci China Life Sci. 2020;63:92–101. 31709495 10.1007/s11427-019-1553-5

[67] Sato Y, Takehisa H, Kamatsuki K, Minami H, Namiki N, Ikawa H, et al. RiceXPro version 3.0: expanding the informatics resource for rice transcriptome. Nucleic Acids Res. 2013;14:D1206–13. 10.1093/nar/gks1125 PMC3531122 23180765

[68] Wu J, Qi Y, Hu G, Li J, Li Z, Zhang H. Genetic architecture of flag leaf length and width in rice (*Oryza sativa* L.) revealed by association mapping. Genes Genomics. 2017;39:341–52.

[69] Li S, Rao Y, Duan P, Wang Z, Hu P, Yu R, et al. Mapping and candidate gene prediction of qPL7-25: a panicle length QTL in Dongxiang Wild Rice. Agriculture. 2023;13:1623.

[70] Liu Z, Sun H, Zhang Y, Du M, Xiang J, Li X, et al. Mining the candidate genes of rice panicle traits via a genome-wide association study. Front Genet. 2023;14:1239550. 37732315 10.3389/fgene.2023.1239550 PMC10507276

[71] Zhong H, Liu S, Meng X, Sun T, Deng Y, Kong W, et al. Uncovering the genetic mechanisms regulating panicle architecture in rice with GPWAS and GWAS. BMC Genomics. 2021;22:1–13. 33509071 10.1186/s12864-021-07391-x PMC7842007

[72] Thapa R, Tabien RE, Septiningsih EM. Genome-wide association study to identify chromosomal regions related to panicle architecture in rice (*Oryza sativa*). Genet Resour Crop Evol. 2021;:1–17.

[73] Du M, Xiong M, Chang Y, Liu Z, Wang R, Lin X, et al. Mining candidate genes and favorable haplotypes for flag leaf shape in rice (*Oryza sativa* L.) based on a genome-wide association study. Agronomy. 2022;12:1814.

[74] Singh G, Khanna R, Kaur R, Kaur K, Kaur R, Sharma N, et al. Performance under multi-environment trial for quantitative traits of rice (*Oryza sativa* L.) genotypes in North-West India (Punjab). Ecol Genet Genomics. 2023;28:100190.

[75] Panda D, Sahu N, Behera PK, Lenka K. Genetic variability of panicle architecture in indigenous rice landraces of Koraput region of Eastern Ghats of India for crop improvement. Physiol Mol Biol Plants. 2020;26:1961–71. 33088042 10.1007/s12298-020-00871-6 PMC7548273

[76] Nakano H, Yoshinaga S, Takai T, Arai-Sanoh Y, Kondo K, Yamamoto T, et al. Quantitative trait loci for large sink capacity enhance rice grain yield under free-air CO2 enrichment conditions. Sci Rep. 2017;7:1827. 28500344 10.1038/s41598-017-01690-8 PMC5431863

[77] Guttikonda H, Chandu G, Munnam SB, Beerelli K, Balakrishnan D, Madhusudhana R, et al. Mapping for yield related traits in rice reveals major effect QTL q*FLA1. 1* from *Oryza Nivara* increases flag leaf area. Euphytica. 2024;220:53.

[78] Adam H, Gutiérrez A, Couderc M, Sabot F, Ntakirutimana F, Serret J, et al. Genomic introgressions from African rice (*Oryza glaberrima*) in Asian rice (*O. sativa*) lead to the identification of key QTLs for panicle architecture. BMC Genomics. 2023;24:587. 37794325 10.1186/s12864-023-09695-6 PMC10548634

[79] Dong H, Zhao H, Li S, Han Z, Hu G, Liu C, et al. Genome-wide association studies reveal that members of bHLH subfamily 16 share a conserved function in regulating flag leaf angle in rice (*Oryza sativa*). PLoS Genet. 2018;14:e1007323. 29617374 10.1371/journal.pgen.1007323 PMC5902044

[80] Wang N, Wang X, Qian Y, Bai D, Bao Y, Zhao X, et al. Genome-Wide Association Analysis of Rice Leaf Traits. Agronomy. 2023;13:2687.

[81] Donde R, Mohapatra S, Baksh SY, Padhy B, Mukherjee M, Roy S, et al. Identification of QTLs for high grain yield and component traits in new plant types of rice. PLoS ONE. 2020;15:e0227785. 32673318 10.1371/journal.pone.0227785 PMC7365460

[82] Xu Z, Miao Y, Chen Z, Gao H, Wang R, Zhao D, et al. Identification and fine mapping of *qGN1c*, a QTL for grain number per panicle, in rice (*Oryza sativa*). Mol Breed. 2019;39:1–12.

[83] Singh G, Pradhan AK, Das Jyoti S, Harper CL, Elumalai P, Samonte SOP et al. Deciphering the Genomic Regions Associated with Seedling Cold Tolerance traits in Rice (*Oryza Sativa* L.). 2024.

[84] Yang J, Yang M, Su L, Zhou D, Huang C, Wang H, et al. Genome-wide association study reveals novel genetic loci contributing to cold tolerance at the germination stage in indica rice. Plant Sci. 2020;301:110669. 33218635 10.1016/j.plantsci.2020.110669

[85] Naveed SA, Zhang F, Zhang J, Zheng TQ, Meng LJ, Pang YL, et al. Identification of QTN and candidate genes for salinity tolerance at the germination and seedling stages in rice by genome-wide association analyses. Sci Rep. 2018;8:6505. 29695843 10.1038/s41598-018-24946-3 PMC5916932

[86] Malumpong C, Cheabu S, Mongkolsiriwatana C, Detpittayanan W, Vanavichit A. Spikelet fertility and heat shock transcription factor (hsf) gene responses to heat stress in tolerant and susceptible rice (*Oryza sativa* L.) genotypes. J Agric Sci. 2019;157:283–99.

[87] Ye J, Yang X, Hu G, Liu Q, Li W, Zhang L, et al. Genome-wide investigation of heat shock transcription factor family in wheat (*Triticum aestivum* L.) and possible roles in anther development. Int J Mol Sci. 2020;21:608. 31963482 10.3390/ijms21020608 PMC7013567

[88] Xu Q, Yu H, Xia S, Cui Y, Yu X, Liu HE, et al. The C2H2 zinc-finger protein LACKING RUDIMENTARY GLUME 1 regulates spikelet development in rice. Sci Bull. 2020;65:753–64. 10.1016/j.scib.2020.01.019 36659109

[89] Sato Y, Antonio B, Namiki N, Motoyama R, Sugimoto K, Takehisa H, et al. Field transcriptome revealed critical developmental and physiological transitions involved in the expression of growth potential in japonica rice. BMC Plant Biol. 2011;11:1–15. 21226959 10.1186/1471-2229-11-10 PMC3031230

